# Reply to Comment on Nogueira, P.J., et al. “The Role of Health Preconditions on COVID-19 Deaths in Portugal: Evidence from Surveillance Data of the First 20293 Infection Cases”. *J. Clin. Med.* 2020, *9*, 2368

**DOI:** 10.3390/jcm9113449

**Published:** 2020-10-27

**Authors:** Paulo Jorge Nogueira, Miguel de Araújo Nobre, Andreia Costa, Ruy M. Ribeiro, Cristina Furtado, Leonor Bacelar Nicolau, Catarina Camarinha, Márcia Luís, Ricardo Abrantes, António Vaz Carneiro

**Affiliations:** 1 IMPSP—Instituto de Medicina Preventiva e Saúde Pública, Faculdade de Medicina, Universidade de Lisboa, Avenida Professor Egas Moniz, 1649-028 Lisboa, Portugal; cristina.furtado@insa.min-saude.pt (C.F.); lnicolau@medicina.ulisboa.pt (L.B.N.); avc@medicina.ulisboa.pt (A.V.C.); 2 Laboratório de Biomatemática, Faculdade de Medicina, Universidade de Lisboa, Avenida Professor Egas Moniz, 1649-028 Lisboa, Portugal; ruyribeiro@medicina.ulisboa.pt; 3 ISBE—Instituto de Saúde Baseada na Evidência, Faculdade de Medicina, Universidade de Lisboa, Avenida Professor Egas Moniz, 1649-028 Lisboa, Portugal; 4 ISAMB—Instituto de Saúde Ambiental, Faculdade de Medicina, Universidade de Lisboa, Avenida Professor Egas Moniz, 1649-028 Lisboa, Portugal; andreiajsilvadacosta@gmail.com; 5 Clínica Universitária de Estomatologia, Faculdade de Medicina, Universidade de Lisboa, Avenida Professor Egas Moniz, 1649-028 Lisboa, Portugal; mnobre@maloclinics.com; 6 UEPID—Unidade de Epidemiologia, Instituto de Medicina Preventiva e Saúde Pública, Faculdade de Medicina, Universidade de Lisboa, Avenida Professor Egas Moniz, 1649-028 Lisboa, Portugal; ccamarinha@medicina.ulisboa.pt (C.C.); marcialuis@campus.ul.pt (M.L.); ricardoabrantes@campus.ul.pt (R.A.); 7 ESEL—Escola Superior de Enfermagem de Lisboa, Polo Calouste Gulbenkian Avenida Prof Egas Moniz, 1600-190 Lisboa, Portugal; 8 CRC-W—Católica Research Centre for Psychological, Family and Social Wellbeing, Universidade Católica Portuguesa, Palma de Cima, 1649-023 Lisboa, Portugal; 9 National Institute of Health Dr. Ricardo Jorge, Av. Padre Cruz, 1600-560 Lisboa, Portugal; 10 Cochrane Portugal, Faculdade de Medicina, Universidade de Lisboa, Avenida Professor Egas Moniz, 1649-028 Lisboa, Portugal

We thank Costa-Santos C., Ribeiro-Vaz I., and Monteiro-Soares [1] for their comment on our article [2].

The database used in our analyses was provided by the Portuguese authorities, as the official dataset available at the beginning of April. We appreciate the issue mentioned by Costa-Santos et al., and for this reason, we were transparent in raising and discussing the point at length in the Discussion section of our article, specifically referring to the potential under-reporting in pre-existing conditions, the existence of missing data, or the lack of significance of diabetes. Moreover, we performed alternative analyses of the data to explore the consistency and sensitivity of the results presented.

It is well recognized that this type of population database often has issues with missing and wrongly coded data [3]; indeed, this may be one reason why public health authorities are reluctant in sharing them. It is very likely that the current version of the database, mentioned by Costa-Santos et al. also includes errors, and it will be very difficult (if not impossible) to untwine bona fide corrections from newly introduced errors. Nevertheless, these databases provide an important snapshot of the situation at the specific moment, and we applaud the Portuguese authorities for making them available to the scientific community.

In the end, we believe that our results reinforce and are by and large consistent with multiple studies, from different regions, as discussed in our paper. We note that even if it were possible (and it is not) to have a fully error-free database—and consequently, the Odds Ratios (OR) values we reported may change somewhat—these specific values would still vary in time with the accumulation of more data and the evolution of the epidemic. For this reason, we welcome further studies refining and analyzing multiple aspects of this pandemic with updated databases.
